# Perioperative Repercussions of Cannabis Use—Implications for GI Endoscopy Sedation

**DOI:** 10.3390/jcm14197028

**Published:** 2025-10-04

**Authors:** Basavana Goudra, Michael Green

**Affiliations:** 1 Department of Anesthesiology and Medicine, Jefferson Surgical Center, Sidney Kimmel Medical College, Jefferson Health, 111 S 11th Street, #7132, Philadelphia, PA 19107, USA; 2 Sidney Kimmel Medical College, Thomas Jefferson University, 1020 Walnut Street, Philadelphia, PA 19107, USA; michael.green2@jefferson.edu

**Keywords:** marijuana, THC, cannabis, endoscopy, colonoscopy, hyperemesis, intoxication

## Abstract

The legalization of cannabis in multiple U.S. states and several other countries, along with its increasing social acceptance across diverse demographic and socioeconomic groups, has led to a growing number of patients presenting for interventional procedures with a history of cannabis use. Although anesthetic and sedation-related implications may be less pronounced than in major surgery, they remain clinically relevant and warrant careful consideration. Key factors include acute intoxication, chronic use, and cannabis use disorder. Cannabis users often require higher—and sometimes unpredictable—doses of propofol and other sedatives. Inhalational use is associated with airway hyperreactivity, increasing the risk of bronchospasm and, in severe cases, life-threatening laryngospasm. Acute intoxication may also impair the patient’s ability to provide informed consent. Cardiovascular manifestations, including tachycardia, hypertension, and an elevated risk of myocardial infarction, may occur depending on the timing and extent of recent cannabis exposure. Although these effects are unlikely to cause major complications during routine screening colonoscopy or diagnostic esophagogastroduodenoscopy, advanced therapeutic procedures may pose significant challenges for sedation providers. This narrative review summarizes the chemistry, pharmacology, and sedation-related implications of cannabis use in patients undergoing sedation requiring interventional procedures, with a specific focus on GI endoscopy.

## 1. Introduction

Over the past few decades, there has been remarkable progress in elucidating endogenous ligands and their corresponding receptors. Among the best characterized are hormones such as estrogen and insulin; neurotransmitters including dopamine and serotonin; and growth factors such as epidermal growth factor, fibroblast growth factor, platelet-derived growth factor, and insulin-like growth factor. The discovery of endogenous opioids, specifically enkephalins and endorphins, which act on opioid receptors, was first reported in 1975 [1]. The gene encoding a cannabinoid receptor (CB1) was cloned in 1990 [2]. Similar to opioid receptors, cannabinoid receptors (CB1 and CB2) are G protein–coupled receptors that play key roles in metabolic regulation, craving, pain modulation, anxiety, bone growth, and immune function [3]. As a result, the therapeutic scope of both natural and synthetic cannabinoid receptor agonists has expanded beyond the management of pain and anxiety. In parallel, the use of Δ9-tetrahydrocannabinol (THC)-containing agents has also increased. One consequence of cannabis legalization or decriminalization in many countries is the growing need to recognize and appropriately manage patients with cannabis exposure in sedation practice. This review aims to highlight the implications of cannabinoid use for anesthesia and sedation during both routine and advanced interventional procedures.

## 2. Chemistry of Cannabis and Their Products

**Figure 1:**
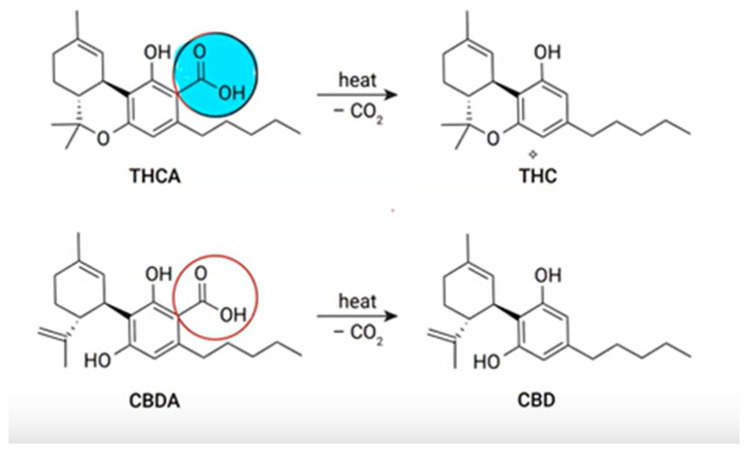
Activation of tetrahydrocannabinolic acid (THCA) and cannabidiolic acid (CBDA) by decarboxylation caused by heat resulting in psychoactive compound THC and non-psychoactive cannabinoid.

Any substance, regardless of its chemical structure or origin, that binds to cannabinoid receptors and elicits effects similar to those of the *Cannabis sativa* plant is referred to as a cannabinoid [4]. The term *endocannabinoids* refers to naturally occurring endogenous ligands, whereas *phytocannabinoids* are 21-carbon compounds derived from the *Cannabis sativa* plant. In their natural state within the *Cannabis* plant, cannabinoids are largely psycho-inactive. To become psychoactive, they must undergo decarboxylation, a process in which the relatively unstable carboxyl group is lost upon heating. Through this reaction, tetrahydrocannabinolic acid (THCa) is converted to THC, and cannabidiolic acid (CBDa) is converted to cannabidiol (CBD). In their carboxylated forms, these compounds are inactive and do not cross the blood–brain barrier. The *Cannabis* plant contains approximately 80 cannabinoids and nearly 300 additional non-cannabinoid compounds, with THC and CBD being the most prominent [5]. While THC possesses strong psychoactive properties and is primarily responsible for the “high” associated with cannabis use, CBD exerts contrasting effects, being widely recognized for its calming, sleep-promoting, and analgesic properties. The molecular structures of THC and CBD, along with their heat-induced activation, are illustrated in Figure 1.

## 3. Pharmacology

Although the terms *cannabis* and *marijuana* are often used interchangeably, they have distinct meanings. *Cannabis* broadly refers to all products derived from the *Cannabis sativa* plant, whereas derivatives containing significant amounts of THC are specifically referred to as *marijuana* [6].

THC-containing products can be administered through multiple routes, including oral ingestion, nasal spray, transdermal application, sublingual drops, and rectal or vaginal suppositories. However, inhalation—via smoking or vaping—remains the most common method of consumption. Cannabis plants contain highly variable concentrations of THC, ranging from approximately 0.3% to 30%, which contributes to significant variability in tissue THC levels following inhalation [7]. Approximately 30% (range: 11–45%) of inhaled THC reaches the systemic circulation, with peak plasma concentrations typically occurring within 10 min of smoking or vaping. By contrast, the oral bioavailability of THC is considerably lower, ranging from 4% to 12%, largely due to extensive first-pass metabolism in the liver. Oral absorption is further influenced by nutritional status—particularly the presence of high-fat meals—as well as by sex-related differences in metabolism and distribution.

Elimination of THC is relatively slow, with trace concentrations detectable for several weeks after use. The majority of cannabis-derived compounds are excreted as metabolites, approximately 65% via feces and about 20% via urine. Metabolism primarily occurs through hydroxylation and carboxylation pathways, and the resulting metabolites are generally considered psycho-inactive [8]. Less than 1% of the administered THC ultimately reaches the brain. When ingested, absorption is highly variable and subject to extensive first-pass metabolism, resulting in reduced and inconsistent systemic availability [9]. Administration via the lungs (inhalation), oral mucosa (oromucosal preparations), or rectum (suppositories) bypasses or reduces the first-pass effect. Among these, inhalation provides the highest bioavailability, followed by oromucosal (including intranasal sprays) and then oral ingestion. In animal studies, the rectal administration of Δ9-THC demonstrated a calculated bioavailability of approximately 13.5% in monkeys [10]. The inferior rectal veins drain into the internal pudendal vein, which subsequently drains into the internal iliac vein and ultimately into the inferior vena cava. This pathway allows rectal administration to bypass hepatic first-pass metabolism. Smoking behaviors, however, vary considerably among individuals, contributing to marked variability in THC bioavailability via the inhalational route. Following inhalation, peak effects typically occur within 6 to 10 min, with THC concentrations in the brain exceeding those measured in the blood.

G protein–coupled receptors (GPCRs), including the cannabinoid receptors CB1 and CB2, represent the principal pathway for signaling in both neuronal and non-neuronal cells. Other important GPCRs include adrenergic receptors, muscarinic acetylcholine receptors, opioid receptors, glucagon-like peptide-1 (GLP-1) receptors, and serotonin receptors.

CB1 receptors are predominantly distributed in the brain, with particularly high concentrations in the basal ganglia, hippocampus, prefrontal cortex, and cerebellum. Beyond the central nervous system, they are also expressed in the enteric nervous system and on the sensory terminals of vagal and spinal neurons, where they play an important role in regulating neurotransmitter release [11]. Their roles in suppressing seizure activity, alleviating pain, mitigating psychosis, and reducing nausea and vomiting, as well as their antioxidant properties, are well documented [12]. CB1 receptors are also involved in the regulation of motor function, as well as in modulating cognition and memory [13]. Activation of CB1 receptors inhibits neurotransmitter release, with a predominance on GABAergic compared to glutamatergic neurons across various brain regions. In contrast, CB2 receptors are primarily expressed in peripheral organs involved in immune function, including macrophages, spleen, tonsils, thymus, and leukocytes, as well as in the lungs and testes. They are also present, though to a lesser extent, in the brain. While both THC and CBD interact with CB1 and CB2 receptors, THC is chiefly responsible for the psychoactive effects of cannabis, whereas CBD is associated with anxiolytic and antipsychotic properties [14]. The adverse effects of THC, as well as its interactions with sedation and anesthesia, are well recognized.

## 4. Extent of Marijuana Use in the USA

According to the 2022 National Survey on Drug Use and Health (NSDUH) conducted by the Substance Abuse and Mental Health Services Administration (SAMHSA), marijuana was reported as the most commonly used illicit drug [15].

Moreover, the 2022 NSDUH reported the following demographic distribution of marijuana use:

Approximately 2.9 million adolescents (5%) aged 12–17 years.

About 13.3 million young adults (22%) aged 18–25 years.

Nearly 45.7 million adults (16%) aged 26 years or older.

Notably, marijuana use disorder was identified in 3 out of every 10 users. In addition, prevalence was higher among men than women, for both recreational and medicinal use.

With respect to ethnicity, in 2022, marijuana use was reported among 31.1% of multiracial individuals. Other groups were American Indian or Alaska Natives (27.3%), Blacks (23.5%), Whites (22.9%), Hispanic (20.3%), and Asians (11.2%). The survey further highlighted concerning trends among adolescents: nearly one-third of 12th graders reported marijuana use in 2021, while an additional 10% reported having experimented with it at least once. Daily use was documented in 6.3% of this group.

Among young adults aged 18–25 years, 38.2% (approximately 13.3 million individuals) reported marijuana use. Overall, marijuana consumption has increased across all age groups following legalization. Pediatric exposures are also concerning; in children aged 10 years and younger, cannabis-related emergency department visits averaged 30.4 to 71.5 per 10,000 weekly visits.

Regarding routes of administration, smoking remained the most common (79.4%), followed by ingestion of edibles (41.6%), vaping (30.3%), and dabbing—defined as inhaling heated, concentrated cannabis extracts—(14.6%). In the 18–24-year age group, vaping and dabbing were particularly prevalent [16].

## 5. Clinical Uses

The therapeutic applications of cannabis have expanded considerably in recent years. Reported indications include the management of emesis, pain, inflammation, multiple sclerosis, anorexia, epilepsy, glaucoma, cancer, obesity, metabolic syndrome–related conditions, Parkinson’s disease, Huntington’s disease, Alzheimer’s disease, and Tourette’s syndrome [17,18,19,20,21,22,23,24,25]. Cannabidiol (CBD), marketed as Epidiolex, has demonstrated therapeutic potential in the management of anxiety, addiction, psychosis, movement disorders, and pain [26,27,28,29,30].

## 6. Sedation Management in Cannabis-Using Patients: Key Challenges

### 6.1. Acute Intoxication

In contrast to patients undergoing major surgical procedures—where perioperative challenges such as pain, postoperative nausea and vomiting (PONV), residual neuromuscular blockade, and surgical complications are common—patients presenting for interventional procedures have markedly different sedation requirements, as well as distinct recovery profiles. The American Society of Regional Anesthesia and Pain Medicine (ASRA) recommends postponing or canceling elective procedures in patients with evidence of acute cannabis intoxication, including altered mental status or impaired decision-making capacity [31]. The clinical features of acute cannabis intoxication, in order of reported frequency, include anxiety (28%), vomiting (24%), agitation (23%), palpitations (14%), reduced consciousness (13%), hallucinations (9%), acute psychosis (9%), chest pain (7%), headache (6%), hypotension (4%), hypertension (3%), and seizures (2%) [32]. Age- and gender-related differences in the clinical manifestations of acute cannabis intoxication have also been reported. Patients younger than 20 years were more likely to present with vomiting (34.7%), reduced consciousness (21.5%), and headache (10.8%), and less likely to develop acute psychosis (5.5%). In contrast, patients older than 49 years more frequently experienced hypotension (6.5%) but less often presented with vomiting (20%), anxiety (14%), agitation (14%), or reduced consciousness (10%).

Gender differences were also observed. Compared with females, males were more likely to present with hypertension (3.7% vs. 1.5%), psychosis (10.4% vs. 6.3%), chest pain (8.1% vs. 4.5%), and seizures (2.5% vs. 1.4%). Conversely, females presented more often with vomiting (28.2% vs. 21.8%), anxiety (32.3% vs. 25.4%), and hypotension (5.8% vs. 2.9%).

From a practical standpoint, important differences exist between alcohol and cannabis intoxication. Unlike alcohol, for which blood alcohol concentration provides a reliable indicator of impairment, there are currently no validated tests to definitively establish cannabis intoxication. Neither field sobriety tests (FSTs) nor measurements of THC levels in blood or other biofluids reliably correlate with the degree of impairment [33]. FSTs alone are insufficient to reliably identify THC-specific driving impairment. Performance on impairment testing varies according to both the dose and the route of administration, and the timing of peak impairment differs with each dosing regimen. Furthermore, biofluid concentrations of THC—whether measured in blood, urine, or oral fluid—do not correlate with FST performance or the degree of intoxication, irrespective of the method of cannabis ingestion. Volunteer studies have also demonstrated that THC biofluid levels fluctuate substantially depending on both the cannabis dose and the route of administration [34].

The behavioral effects of cannabis typically appear within minutes of smoking but occur more gradually with other routes of administration, and may persist for several hours [35]. Notable behavioral effects of cannabis use include euphoria or a subjective “high,” reduced anxiety and depression, and increased sociability. Conversely, some individuals experience opposite reactions such as heightened anxiety, irritability, and, in more severe cases, panic, paranoia, or psychosis. Physical signs of intoxication include conjunctival injection (“red eye”), resulting from vasodilation, and xerostomia (dry mouth), which arises through a similar mechanism. Tachycardia is also commonly observed. Cannabis use is well known to increase appetite, which may lead to a full stomach and, consequently, an elevated risk of aspiration during procedural sedation [35].

Overt signs and symptoms of acute cannabis intoxication are uncommon in clinical practice. However, if a patient demonstrates impaired capacity to provide informed consent for a procedure or anesthesia, the intervention should be postponed or, if necessary, canceled. It is important to note that such levels of intoxication are rarely encountered in anesthesia practice involving interventional procedures such as GI endoscopy.

### 6.2. Challenges with Sedation Administration

Several areas of concern arise when administering intraprocedural sedation to patients who use cannabis. These include altered sedation requirements, potential cardiovascular complications, pulmonary risks, and an increased likelihood of aspiration.

#### 6.2.1. Sedation Requirements

In a case–control study evaluating patients undergoing gastrointestinal endoscopy, Imasogie et al. examined the association between self-reported cannabis use and propofol requirements. Among 318 patients (151 cases and 167 controls), daily cannabis users required significantly higher doses of propofol compared with weekly or monthly users, even after adjusting for potential confounders such as age, sex, weight, ASA physical status, respiratory disease, tobacco use, opioid intake, alcohol consumption, and chronic pain. On average, propofol requirements were increased by 75.98 mg in daily cannabis users. Furthermore, procedural sedation-related challenges were observed more frequently in the cannabis group, including greater oxygen requirements and the need for bag–mask ventilation with oral airway insertion [36]. The procedures evaluated in this study included EGD, colonoscopy, and combined EGD with colonoscopy.

In a retrospective cohort study of subjects undergoing deep sedation or general anesthesia, of 189 patients who met the inclusion criteria, 57 reported using cannabis. The classification was binary, users and non-users. There was no mention of type, amount, duration, frequency, and mode of cannabis intake. The concluded that cannabis users required more propofol, midazolam, ketamine, and fentanyl than non-cannabis users while undergoing outpatient oral and maxillofacial surgical procedures [37].

In a prospective, randomized, single-blinded study of 30 male cannabis users (more than once per week) and 30 nonusers, all aged 18–50 years, the 50% effective dose (ED50) of propofol for induction was assessed. Induction was defined as loss of consciousness with a bispectral index (BIS) value <60 and satisfactory laryngeal mask airway (LMA) insertion. The investigators reported no significant difference in BIS values between groups. However, cannabis users required a significantly more propofol to achieve successful LMA insertion [38]. A limitation of the study is the lack of information on the amount, type, and route of cannabis consumption. Both the dose and timing of last cannabis use are likely to influence anesthetic requirements. Furthermore, cannabis smoking may have a greater impact on propofol requirements due to airway irritability and associated respiratory changes. These factors merit consideration in future studies to better define the interaction between cannabis exposure and anesthetic drug requirements.

In a systematic review and meta-analysis, Bornemann-Cimenti et al. examined the relationship between cannabinoid use and propofol requirements. A total of 11 studies met the inclusion criteria, comprising 4199 patients (1257 cannabinoid users and 2942 controls). The authors reported that cannabinoid users required a significantly higher dose of propofol compared with nonusers, with a mean increase of 23.9 mg (95% CI, 17.8–30.0; *p* < 0.001) in the fixed-effects model and 36.1 mg (95% CI, 18.1–54.1; *p* < 0.001) in the random-effects model [39]. The only study that involved endoscopy in their analysis was of Imasogie et al., that is discussed above.

In another meta-analysis that included eight eligible studies with a total of 2268 patients, cannabis users were found to require significantly higher doses of propofol compared with nonusers. On average, they required an additional 47.3 mg of propofol for induction [40]. The additional propofol requirement was greater in cannabis users undergoing sedation for endoscopic procedures compared with those receiving general anesthesia for surgical procedures (53.0 mg vs. 30.6 mg, respectively).

As mentioned, cannabis use has also been associated with increased requirements for sedatives other than propofol. In patients undergoing gastroscopy, cannabis users had higher odds of requiring greater total sedation, defined as the administration of more than 5 mg of midazolam, more than 100 µg of fentanyl, or the need for adjunctive diphenhydramine [41]. The study was conducted at the Snyder Institute for Chronic Diseases, Cumming School of Medicine, University of Calgary. In Canada, conscious sedation is commonly practiced for endoscopic procedures. This typically involves the administration of sedatives other than propofol by nursing staff under the supervision of the endoscopist performing the procedure.

The mechanisms underlying the increased propofol requirements in cannabis users are not fully understood. Possible explanations include altered central nervous system sensitivity and receptor-level adaptations resulting from chronic cannabis exposure. Several variables likely influence sedative requirements, including the amount, duration, frequency, and route of cannabis intake. Furthermore, the heterogeneity of cannabis strains—with varying concentrations of THC and other cannabinoids—makes it difficult to quantify actual exposure. An additional challenge is the underreporting of cannabis use, as stigma may persist despite legalization, potentially limiting the accuracy of patient disclosure [42].

#### 6.2.2. Cardiovascular Adverse Events

In a population-based, cross-sectional study, Jeffers et al. investigated the association between cannabis use and cardiovascular outcomes in the general population. The analysis also included subgroup evaluations of individuals who had never used tobacco as well as younger participants [43]. They concluded that cannabis use is associated with adverse cardiovascular outcomes, with stronger associations observed among heavier users (greater number of days per month). In this study, cannabis exposure and cardiovascular outcomes were based on self-reported data. The adjusted odds ratios (OR) for the association between daily cannabis use and specific cardiovascular outcomes were as follows:

Coronary heart disease: OR 1.16 (95% CI, 0.98–1.38)

Myocardial infarction: OR 1.25 (95% CI, 1.07–1.46)

Stroke: OR 1.42 (95% CI, 1.20–1.68)

Composite outcome (coronary heart disease, myocardial infarction, and stroke): OR 1.28 (95% CI, 1.13–1.44)

The increased cardiovascular risk associated with cannabis use was observed in men under 55 years and women under 65 years. Notably, individuals who had never used tobacco also demonstrated higher risks for all three complications. However, these associations primarily indicate a greater likelihood of encountering cardiovascular risk factors in patients presenting for gastrointestinal endoscopy; they do not, by themselves, imply an increased perioperative risk.

In contrast, Goel et al., in a retrospective population-based cohort study of patients undergoing elective surgery, reported that active cannabis use disorder was associated with a significantly higher risk of postoperative myocardial infarction, with an adjusted odds ratio of 1.88 (95% CI, 1.31–2.69; *p* < 0.001). The composite postoperative complication rate, however, was not significantly different between patients with (2.9%) and without (3.1%) cannabis use disorder. Cannabis use disorder is broadly defined as the inability to stop consuming cannabis despite experiencing physical or psychological harm [44].

An exploratory study examined the relationship of marijuana use with coronary heart disease [45]. In an inception cohort study of 1913 adults hospitalized with myocardial infarction across centers in the USA, there were six deaths among self-reported marijuana users compared with one death among non-users. Mittleman et al. interviewed 3882 patients (including 1258 women) with acute myocardial infarction, on average four days after infarction onset. They found that the risk of triggering a myocardial infarction was elevated 4.8-fold within one hour of smoking marijuana compared with periods of nonuse. Interestingly, marijuana users were less likely to have a prior history of angina (12% vs. 25%, *p* < 0.001) or hypertension [46].

After conducting a systematic review to evaluate the acute cardiovascular effects of marijuana use, Ghasemiesfe et al. found that 14 studies, including seven randomized controlled trials, reported an increase in heart rate following marijuana exposure. However, the risk of bias varied across the included studies [47]. The effects of marijuana on blood pressure were variable. One randomized controlled trial reported a reduction in cerebral blood velocity, whereas another found no change in global cerebral blood flow. In a separate systematic review, Ravi et al. concluded that there was insufficient evidence to establish a definitive association between marijuana use and cardiovascular risk factors or outcomes, including stroke and myocardial infarction [48]. Amsterdam et al. analyzed 22 eligible publications to evaluate the relationship between cannabis use and myocardial infarction. They concluded that frequent and current cannabis use in forms other than smoking—such as vaping or ingestion—was not associated with a significantly increased cardiovascular risk [49]. Furthermore, Amsterdam et al. noted that, similar to tobacco smoking, cannabis smoking may independently trigger myocardial infarction. In contrast, Jeffers et al., in a study published in *JAMA* in 2024, reported that daily cannabis users had 25% higher odds of myocardial infarction and 42% higher odds of stroke compared with nonusers.

In conclusion, although chronic marijuana use is associated with increased morbidity and mortality from myocardial infarction, these findings should not be extrapolated to gastrointestinal endoscopic procedures. There is little evidence to justify canceling or delaying endoscopic procedures solely on the basis of same-day or recent (within the preceding four hours) cannabis use from a cardiovascular risk perspective.

#### 6.2.3. Pulmonary Adverse Events

From a pulmonary standpoint, there is a prevailing perception in the USA that daily cannabis smoking and exposure to second-hand cannabis smoke are safer than tobacco smoke [50,51]. Nevertheless, both cannabis and tobacco smoke are toxic mixtures of gases and particulates that are harmful to health. Janssen et al. reported that, compared with a reference cigarette, cannabis smoke emitted 105% more fine particulate matter with a diameter of less than 1 μm (PM1) and 93% more particles with diameters ≤ 10 μm (PM10) [52]. In a survey of a random sample of 878 individuals aged 40 years or older, Tan et al., reporting from Vancouver, Canada, found that tobacco smokers reported respiratory symptoms more frequently than marijuana smokers. Additionally, concurrent use of marijuana and tobacco was associated with a higher risk of respiratory symptoms and chronic obstructive pulmonary disease (COPD), after adjusting for age, asthma, and comorbidities, compared with tobacco smoking alone. The participants’ lifetime marijuana exposure exceeded 50 marijuana cigarettes [53].

Some long-term effects of marijuana smoking include chronic cough, sputum production, histopathologic evidence of widespread airway inflammation and injury, and immunohistochemical signs of dysregulated growth of respiratory epithelial cells, which may represent precursors to lung cancer. THC has been shown to increase oxidative stress, cause mitochondrial dysfunction, and inhibit apoptosis. Nevertheless, there is no conclusive evidence that marijuana smoking directly causes chronic obstructive pulmonary disease or lung cancer. Similarly, current data are insufficient to establish a causal link between cannabis smoking and bullous lung disease or spontaneous pneumothorax [54].

Using data from the UK Biobank, which included 500,000 individuals (229,134 men and 273,402 women), predominantly of White ethnicity, Lehrer et al. were unable to determine the independent adverse effects of cannabis smoking separate from tobacco use. Nevertheless, they acknowledged that cannabis use has the potential to cause severe lung damage [55]. In a similar study, neither former nor current marijuana smoking, regardless of lifetime exposure, was associated with the development or progression of COPD [56].

As noted earlier in this review, cannabis smokers may require higher doses of sedative agents, including propofol. One contributing factor may be increased airway irritability. It is recognized that endotracheal intubation can be more challenging in marijuana smokers, and airway manipulation may precipitate wheezing and coughing [57,58]. Recent marijuana use has been associated with lower levels of exhaled nitric oxide and higher forced vital capacity, a pattern similar to that observed with recent tobacco smoking [59].

#### 6.2.4. Gastric Emptying

Cannabinoid receptors are expressed throughout the gastrointestinal tract, where they regulate peristalsis and intestinal barrier permeability. Both THC and cannabidiol (CBD) influence gut motility primarily via the CB1 receptor. Consequently, cannabinoids are being investigated for their potential therapeutic role in managing gastrointestinal motility disorders [60].

In a randomized, double-blind crossover study of 13 healthy volunteers, McCallum et al. demonstrated that administration of THC significantly delayed gastric emptying of solid food compared with placebo [61]. THC was administered at a dose of 10 mg/m^2^ of body surface area, and no correlation was observed between plasma THC levels and the delay in gastric emptying. For reference, marijuana joints (hand-rolled cannabis cigarettes) typically contain a median of 6.56 mg of Δ9-THC and 0.02 mg of CBD, whereas hashish joints contain a median of 7.94 mg of Δ9-THC and 3.24 mg of CBD. A joint is made exclusively from cannabis flower and rolling paper [62].

A 24-year-old man with a body mass index of 22 regurgitated approximately 150 mL of gastric contents shortly after LMA insertion [63]. The patient was scheduled for open reduction and internal fixation of a right lower extremity fracture. He reported daily marijuana use (approximately 2 g/day for six years) but had fasted according to standard guidelines. Despite this, he regurgitated gastric contents shortly after LMA insertion. Rapid endotracheal intubation was performed without adverse events; however, the case highlights the potential for large residual gastric volumes in chronic cannabis users despite adherence to fasting protocols. Following intubation, the anesthesia provider suctioned 500 mL of clear gastric contents from the stomach. The patient denied any history of cannabis hyperemesis or recent nausea.

The non-selective cannabinoid agonist dronabinol has been shown to slow gastric emptying. It is also known to inhibit colonic tone and phasic pressure activity [64]. Gastrointestinal and colonic dysmotility associated with cannabinoids has been demonstrated in both human and animal models.

#### 6.2.5. Emergence from Anesthesia

In a retrospective analysis from a single center (Spine Care Institute, Hospital for Special Surgery, New York, NY, USA), Amoroso examined 204 patients who had undergone single- or multilevel spinal fusion surgery [65]. The study focused on the relationship between self-reported cannabis use, patient demographics, surgical characteristics, numeric rating scale (NRS) pain scores, anxiety, inpatient opioid consumption, and discharge prescribing (total morphine equivalent dose). As noted previously, stigma associated with cannabis use may prevent patients from fully disclosing their consumption, representing a major limitation of studies relying on self-reported data. In their analysis, cannabis use was associated with higher preoperative, but not necessarily postoperative, pain scores. Postoperative opioid consumption among cannabis users did not differ significantly from nonusers.

It is, however, well documented that daily cannabis users often experience higher postoperative pain scores—though this is less relevant for gastrointestinal endoscopy, as these patients do not typically experience the same degree of surgical pain—and may require interventions including opioid administration. Interestingly, animal studies suggest that cannabinoids can augment the analgesic effects of opioids. This effect is likely mediated by synergistic interactions between the endogenous cannabinoid system and the endogenous opioid system at both the spinal cord and central nervous system levels [66].

In patients exhibiting signs and symptoms of acute cannabis intoxication, emergence from anesthesia may be less smooth [58]. Fever, tachycardia, and hypertension may be observed during the recovery period and could raise suspicion for conditions such as malignant hyperthermia, serotonin syndrome, neuroleptic malignant syndrome, or 3,4-methylenedioxymethamphetamine (MDMA) overdose.

In a retrospective cohort study of 27,388 patients conducted at two academic medical centers—the University of Washington Medical Center and Harborview Medical Center, USA—all patients (ASA 1–3) underwent non-obstetric, non-cardiac procedures under general anesthesia. Cannabis use and postoperative nausea and vomiting (PONV) in the post-anesthesia care unit (PACU) were documented. The analysis demonstrated an increased relative risk and a modest increase in the marginal probability of PONV among cannabis users [67]. It should be noted, however, that cannabis-based medicines are also used therapeutically to treat nausea and vomiting, particularly in the context of chemotherapy-induced symptom [68,69,70].

Postoperative shivering has been reported to occur more frequently among cannabis users. In a prospective, cross-sectional observational study, Sankar-Maharaj et al. evaluated 55 patients and found an overall incidence of postoperative shivering of 36%. Among cannabis users (25/55), the incidence was 40%, compared with 33.3% in non-users [71].

#### 6.2.6. Cannabinoid Hyperemesis Syndrome

Cannabinoid hyperemesis syndrome (CHS) is characterized by cyclic episodes of nausea and vomiting, typically occurring in chronic cannabis users. Symptoms often improve with hot baths. Since the legalization of cannabis in Colorado, the frequency of patients presenting to emergency departments for CHS has reportedly doubled [72]. The exact mechanism of CHS remains unknown, and traditional antiemetics are often ineffective. CHS is classically described in three phases: prodromal, hyperemetic, and recovery [73]. The prodromal, or preemetic, phase of cannabinoid hyperemesis syndrome (CHS) is characterized by early-morning nausea without vomiting, often accompanied by abdominal discomfort. The hyperemetic phase typically presents with intractable nausea and vomiting, which may be associated with mild, diffuse abdominal pain, and is often the reason for emergency room visits [72].

In a retrospective observational cohort study utilizing electronic health records from Kaiser Permanente Northern California, Costales et al. reported a substantial increase in emergency department visits for CHS [74]. Similar observations have been reported by Habboush and Soh et al. [75,76].

The clinical significance of CHS in the context of gastrointestinal endoscopy is limited. A history of CHS indicates chronic cannabis use or potentially cannabis use disorder, which may prompt providers to further evaluate the patient and remain cognizant of associated considerations, such as cannabis withdrawal. For the majority of endoscopic procedures, CHS is unlikely to interfere. However, in complex or advanced procedures that require general anesthesia and have prolonged duration, these factors may be more relevant and could influence perioperative management.

#### 6.2.7. Relevant Drug Interactions

THC inhibits the CYP2C9-mediated metabolism of warfarin [77]. Yamreudeewong et al. reported a case of a 56-year-old White male on long-term warfarin therapy (11 years) following mechanical heart valve replacement, who presented with upper gastrointestinal bleeding. While concurrently smoking marijuana, his international normalized ratio (INR) was markedly elevated, measuring 10.41 on one occasion and 11.55 on another [78]. After discontinuation of marijuana, the patient’s INR values returned to the 1.08–4.40 range. In addition to inhibiting CYP2C9, marijuana may also displace warfarin from its plasma protein-binding sites. Several other case reports have documented potential drug interactions between cannabinoids and oral anticoagulants [79].

Cannabidiol is also known to inhibit CYP2C19, an isoenzyme responsible for the transformation of clopidogrel to its active thiol metabolite. As a result, the therapeutic efficacy could be reduced [80].

Table 1 contains a summary of important implications of various perioperative attributes of cannabis use.

## 7. Conclusions

With the legalization of recreational marijuana, gastroenterologists and anesthesia providers are increasingly likely to encounter patients who use cannabis. Despite legalization, stigma persists, and many patients may be reluctant to disclose marijuana use unless the rationale for doing so is clearly explained. Simply mentioning potential anesthesia interactions is often sufficient to obtain an accurate history.

Currently, there is insufficient evidence to justify delaying or canceling routine gastrointestinal endoscopic procedures, even if the patient has consumed marijuana a few hours prior. Sedation requirements in cannabis users may be higher, although findings are not entirely consistent. Cardiovascular perturbations, including an elevated risk of myocardial infarction, are unlikely to be clinically significant during endoscopic procedures. Emergence from sedation may occasionally be less smooth, but this is generally not concerning.

Potential interactions with warfarin warrant checking the INR before interventions that carry bleeding risk. Infrequently, patients present for gastrointestinal complications related to chronic marijuana use, including cannabinoid hyperemesis syndrome. Patients who are acutely intoxicated to the extent that they cannot provide valid informed consent should have their procedure deferred.

## Figures and Tables

**Table 1:** Anesthesia and sedation considerations in patients using cannabis.

Attribute	Implication/s	References	Notes
Sedation requirements	Increased requirements for propofol, midazolam, ketamine, and fentanyl Greater oxygen requirements, increased need for bag–mask ventilation with oral airway insertion Higher propofol dose to achieve successful LMA insertion	[36,37,38,39,40,41,42]	Related to airway irritation (LMA insertion), altered central nervous system sensitivity and receptor-level adaptations. Related to the amount, duration, frequency, and route of cannabis intake
Cardiovascular adverse events	Increased risk of adverse cardiovascular events including CHD, MI. Stroke; related to amount and duration of exposure. Risk of triggering a myocardial infarction elevated 4.8-fold within one hour of smoking marijuana compared with periods of nonuse.	[43,44,45,46,47,48,49]	Supporting evidence is mainly retrospective. Currently insufficient evidence to cancel or delay a procedure based on history of cannabis use
Pulmonary adverse events	Concurrent use of marijuana and tobacco associated with a higher risk of respiratory symptoms and COPD, after adjusting for age, asthma, and comorbidities, compared with tobacco smoking alone. Long-term effects include chronic cough, sputum production, histopathologic evidence of widespread airway inflammation and injury	[50,51,52,53,54,55,56,57,58,59]	A definitive cause effect relationship between cannabis smoking and COPD is lacking
Gastric emptying	THC significantly delays gastric emptying of solid food compared with placebo.	[60,61,62,63,64]	Insufficient evidence to make definitive recommendations
Emergence from anesthesia	Daily cannabis users often experience higher postoperative pain scores. In those showing features of acute cannabis intoxication, emergence is delayed and less smooth. Postoperative shivering is more common among cannabis users.	[65,66,67,68,69,70,71]	Supporting evidence is mainly retrospective
Cannabinoid hyperemesis syndrome	Mainly seen in ER. A history of CHS indicates chronic cannabis use. CHS might be an indication for EGD.	[72,73,74,75,76]	CHS does not respond to standard antiemetics
Drug Interactions	THC inhibits the CYP2C9-mediated metabolism of warfarin. Cannabidiol inhibits an isoenzyme responsible for the transformation of clopidogrel to its active thiol metabolite	[77,78,79,80]	

CHD—coronary artery disease, MI—myocardial infarction, ER—emergency room, CHS—cannabinoid hyperemesis syndrome, EGD—esophagogastroduodenoscopy, THC—delta-9-tetrahydrocannabinol, CYP2C9—cytochrome P450 family 2 subfamily C member 9.

## Data Availability

Not applicable.
